# Variation in the Circumsporozoite Protein of *Plasmodium falciparum*: Vaccine Development Implications

**DOI:** 10.1371/journal.pone.0101783

**Published:** 2014-07-03

**Authors:** Kavita Gandhi, Mahamadou A. Thera, Drissa Coulibaly, Karim Traoré, Ando B. Guindo, Amed Ouattara, Shannon Takala-Harrison, Andrea A. Berry, Ogobara K. Doumbo, Christopher V. Plowe

**Affiliations:** 1 Howard Hughes Medical Institute/Center for Vaccine Development, University of Maryland School of Medicine, Baltimore, Maryland, United States of America; 2 Malaria Research and Training Center, University of Science, Techniques and Technology, Bamako, Mali; London School of Hygiene and Tropical Medicine, United Kingdom

## Abstract

The malaria vaccine candidate RTS,S/AS01 is based on immunogenic regions of *Plasmodium falciparum* circumsporozoite protein (CSP) from the 3D7 reference strain and has shown modest efficacy against clinical disease in African children. It remains unclear what aspect(s) of the immune response elicited by this vaccine are protective. The goals of this study were to measure diversity in immunogenic regions of CSP, and to identify associations between polymorphism in CSP and the risk of *P. falciparum* infection and clinical disease. The present study includes data and samples from a prospective cohort study designed to measure incidence of malaria infection and disease in children in Bandiagara, Mali. A total of 769 parasite-positive blood samples corresponding to both acute clinical malaria episodes and asymptomatic infections experienced by 100 children were included in the study. Non-synonymous SNP data were generated by 454 sequencing for the T-cell epitopes, and repeat length data were generated for the B-cell epitopes of the *cs* gene. Cox proportional hazards models were used to determine the effect of sequence variation in consecutive infections occurring within individuals on the time to new infection and new clinical malaria episode. Diversity in the T-cell epitope-encoding regions Th2R and Th3R remained stable throughout seasons, between age groups and between clinical and asymptomatic infections with the exception of a higher proportion of 3D7 haplotypes found in the oldest age group. No associations between sequence variation and hazard of infection or clinical malaria were detected. The lack of association between sequence variation and hazard of infection or clinical malaria suggests that naturally acquired immunity to CSP may not be allele-specific.

## Introduction

To date, malaria vaccine development and testing has generally not been informed by molecular epidemiologic evaluations of how genetic diversity in vaccine antigens in parasite populations may affect vaccine efficacy. For example, vaccines that confer allele-specific protection may not be effective in a parasite population in which the vaccine allele is rare, and may create a selective advantage favoring non-vaccine alleles, compromising vaccine efficacy [1]. Furthermore, studies of naturally acquired immunity to two *Plasmodium falciparum* blood stage antigens found that immune responses to these antigens may be allele-specific, and a field trial of a vaccine based on one of these antigens reported allele-specific efficacy [2]–[5].

The malaria vaccine RTS,S/AS01 targets specific immunogenic epitopes of the *P. falciparum* circumsporozoite protein (CSP), encoded by the *cs* gene. The *cs* gene is polymorphic, with diversity in regions that encode epitopes recognized by the human immune system. The central repeat region of the *cs* gene contains tetrameric repeats that vary in both the sequence and number of tetramers. This region codes for epitopes recognized by anti-CSP antibodies [6], [7]. It has been suggested that the length of the repeat region may play a role in the stability of the protein, and may therefore affect how B-cell epitopes are displayed to the immune system [8]. The 3′ regions of the *cs* gene, Th2R and Th3R, encode epitopes that are recognized by CD8+ and CD4+ T-cells [9]. The diversity in these regions, which occurs in the form of non-synonymous SNPs, increases as malaria transmission increases across distinct geographic areas [10], [11], with the highest diversity occurring in Africa. Molecular surveys in Sierra Leone and the Gambia found 42 haplotypes in 99 samples and 24 haplotypes in 44 samples for the region containing Th2R and Th3R respectively [10], [12]. RTS,S/AS01 has shown modest efficacy in Phase 2 trials [13]–[16] and a Phase 3 trial [17], [18].

Follow-up studies to Phase 2 trials of the vaccine evaluated evidence of selection of non-vaccine strains in vaccinated vs. non-vaccinated study participants. In a Phase 2 trial of RTS,S in children in Mozambique no evidence of selection favoring non-vaccine strains was found [19]. A follow-up study to a Phase 2 vaccine trial in Kenyan adults identified two polymorphic sites at which a statistically different proportion of non-vaccine strain alleles was found between control and vaccine groups [20]. However, as the selection effects were in opposite directions the authors concluded that there was no evidence of vaccine selection.

Both of these studies used direct sequencing to detect polymorphism in the regions coding for the T-cell epitopes, Th2R and Th3R, and excluded samples that could not be resolved into predominant alleles from the analysis. Moreover, diversity in the central repeat region of the *cs* gene which encodes the B-cell epitopes of CSP and which is also included in the vaccine, was not considered.

To design more effective malaria vaccines and to help interpret efficacy data from vaccine trials, an understanding of the dynamics of polymorphism in vaccine antigens and the factors that are driving that polymorphism is necessary. With the aid of next generation sequencing to help circumvent some of the issues encountered by previous studies of genetic diversity in CSP, this study has examined both the population level and within-host dynamics of polymorphism in this vaccine antigen. We have previously shown that in consecutive infections experienced by Malian children followed in a prospective cohort study, changes at specific polymorphic sites, but not at other polymorphic sites, within two blood-stage *P. falciparum* antigens, were associated with a higher risk of clinical malaria [2], [3]. These findings are taken to indicate that infections “look immunologically different” at these polymorphic amino acid positions and therefore are more likely to result in clinical disease, whereas infections that differ from a previous infection only at amino acid positions that do not generate allele-specific immunity are better tolerated and are less likely to manifest clinical symptoms. Here we used a similar approach to determine whether polymorphism in CSP were associated with allele-specific immunity akin to that seen with the blood-stage vaccine antigens merozoite surface protein 1 and apical membrane antigen 1.

## Materials and Methods

### Ethics statement

The study was approved by the institutional review boards of the University of Sciences, Techniques and Technology Faculty of Medicine, Pharmacy and Dentistry in Bamako, Mali and the University of Maryland, Baltimore. Informed consent was obtained from all study participants or their guardians. The trial was conducted in compliance with the International Conference on Harmonization of Good Clinical Practices, the Declaration of Helsinki, and regulatory requirements of Mali.

### Parent study description

The study was conducted in Bandiagara, a rural town of approximately 13,000 inhabitants located in central Mali. Transmission of *falciparum* malaria is intense with the peak coinciding with the rainy season from July–October. Children aged less than 10 years experience, on average, two clinical episodes a year, and the prevalence of parasitemia at the beginning of the study before the onset of malaria transmission was 17% [21].

A complete population census was conducted in Bandiagara before study initiation. Study participants were sampled in proportion to the population size in each of the eight districts comprising the town of Bandiagara. Study subjects were aged ≥3 months to 20 years. Recruitment of study subjects took place in randomly selected households until the target number of subjects in each age group was achieved.

The study was conducted prospectively during the years 1999, 2000, and 2001. From July to January of each year, blood samples were collected on 3 MM Whatman filter paper monthly and at every episode of clinical malaria. Clinical malaria episodes were detected both by passive surveillance through provision of around-the-clock free clinical care, as well as by active weekly follow-up of the children in the study by physicians working at the study site. Clinical episodes were defined as blood smears positive for *P. falciparum* asexual parasites and symptoms consistent with malaria including, fever, anemia, headache, body aches, cough, diarrhea, or abdominal pain, in the absence of any other obvious cause of symptoms. Infections were defined as the presence of *falciparum* parasites in the blood, with or without symptoms [21].

### Present study description

One hundred children with at least two years of follow up during the malaria incidence study were chosen [2]. These children were randomly selected within three age strata. Thirty children aged ≤5 years, 32 children aged 6 to 10 years, and 38 children aged ≥11 years were selected. Blood samples (n = 2309) corresponding to all monthly surveys (n = 1801) and clinical episodes (n = 508) occurring during the transmission season of the three years of the incidence study underwent DNA extraction (QIAamp DNA Mini Kit, Qiagen, Valencia, California). Of these, 769 parasite-positive samples were subjected to sequencing analysis.

### PCR amplification

The primary forward and reverse PCR primers were GTTGAGGCCTTTTCCAGGAATACCAG and GTACAACTCAAACTAAGATGTGTTC. These primers were designed to amplify the region of the *cs* gene that contained both the repeat region and the Th regions. Primary PCR conditions are as follows: 30 cycles of 95°C for 30 s, 52°C for 30 s, 72°C for 1 min. The secondary forward and reverse PCR primers for the repeat region were TGGGAAACAGGAAAATTGG and GCACTGTTGGCATTAGCATTT. Secondary PCR conditions for the repeat regions and ThRs were 30 cycles of 95°C for 30 s, 55°C for 30 s, 72°C for 1 min 30 s, and 25 cycles of 95°C for 30 s, 58°C for 30 s, and 72°C for 1 min respectively. PCR products were amplified using HotStar Taq (Qiagen, Valencia, California). Secondary PCR primers for the ThRs contained specific adapters necessary for the emPCR [22] step of 454 sequencing, as well as unique barcodes 10–12 bp in length, to identify sequences from individual samples. Sequences for adapter A and B are as follows: CGTATCGCCTCCCTCGCGCCATCAG and CTATGCGCCTTGCCAGCCCGCTCAG. Sequence specific primers for the Th and repeat regions were previously reported in a methods paper [23]. A total of 96 primers containing 96 unique barcode sequences were used to amplify this region from study samples. Primers were identical with the exception of the barcode sequence.

The concentration of each PCR product containing Th2R and Th3R was determined by band intensity measurements taken on the Qiaxcel capillary gel imaging system (Qiagen, Valencia, California), and 100 ng of each product was then pooled. Identical barcodes were used to tag more than one sample. PCR products were separated into sixteen separate pools containing approximately 45 samples each ensuring that no pool contained more than one sample with the same barcode. Each pool was physically separated within a 454 sequencing run. Pooled PCR products were sequenced at the University of Maryland School of Medicine Genomic Resource Center at the Institute of Genome Sciences on the GS FLX Titanium 454 Platform (Roche Diagnostics, Branford, CT). Sequences were aligned using gsAmplicon (Roche Diagnostics, Branford, CT) software. For samples containing more than one allele at a polymorphic site, predominance was determined if the majority allele was present in 71% or more of all reads obtained for that sample. This cut-off was determined in the methods validation study performed by the same authors comparing 454 and Sanger sequencing [23]. If a majority allele could not be determined, that polymorphic site was considered polyclonal. Haplotype information, however, was still obtained for samples with polyclonal polymorphic sites, because complete sequence reads were available for each variant detected. Only haplotypes representing at least 10% of all 454 reads obtained for a sample were considered for analysis.

The methods validation study established the comparability of these methods with direct sequencing as well as comparable majority/minority allele cut-off values. The results of that study indicated that 454 was more sensitive at detecting minor alleles and more accurate in the quantitation of these alleles than direct sequencing [23].

### Determination of repeat region length

To determine the length of the repeat region, PCR products were run on a high resolution gel cartridge on a Qiaxcel capillary gel imaging system (Qiagen, Valencia, California) using the OM500 analysis method capable of resolving size within 10 base pairs (bp). To validate this method, a 96-well plate containing 15 ul of repeat region PCR product amplified from 100 ng/ul 3D7 genomic DNA per well, was run using the method listed above. A systematic underestimation of 10 bp was observed in the experimental data. A 10 bp correction was therefore applied to length values obtained for PCR products from study samples.

Repeat region PCR products for samples that were determined to be ‘single clone’ (no secondary allele present in a frequency greater than 20% in 454 reads) with respect to Th2R and Th3R, were subjected to Sanger sequencing. Once amplification was verified by gel electrophoresis, PCR Products were purified by vacuum filtration in Excela Pure (Edge Biosystems, Gaithersburg, MD) 96-well plates. Purified PCR product was then sequenced on an ABI3730 xl at the University of Maryland School of Medicine Biopolymer Lab.

Sequences were viewed in Sequencher (Gene Codes Corp, Ann Arbor, MI), and then copied to Transeq web software [24] to determine the amino acid sequence and length of the repeat region. Size determinations made via the Qiaxcel for these ‘single clone’ samples were checked against corresponding Sanger sequencing data.

### Determination of mixed infections

Mixed infections with respect to the ThRs were defined as samples with no clear majority allele at a given polymorphic site. Mixed infections with respect to the repeat regions were defined as samples which contained more than one clear band in high-resolution gel analysis.

### Statistical methods

Fisher’s exact tests were used to compare haplotype frequencies between seasons, age groups, and between clinical and non-clinical episodes. Cox proportional hazards models were used to infer the relationship between diversity in Th2R and Th3R and the development of naturally acquired immunity in the context of symptomatic (clinical) and asymptomatic parasitemia (infection). In the clinical model, the association between changes at polymorphic sites in Th2R and Th3R which occurred between consecutive clinical episodes and the hazard of clinical disease was examined. In the infection model, the association between these changes which occurred between a clinical episode and a consecutive asymptomatic infection was examined. Intervals starting with an asymptomatic episode were included in the model if there was an intermediate time point at which the study participant was parasite-negative verified by microscopy. To account for the possibility of treatment failure and to allow for time for allele-specific antibodies to the first of two paired consecutive infections to be present by the time of the second infection, time intervals two weeks or less between consecutive episodes were excluded from the analyses. A logistic regression analysis was performed to determine the odds of a change in the predominant amino acid at polymorphic residues in intervals including an asymptomatic episode followed by a symptomatic one, to intervals including consecutive asymptomatic episodes. Age and time between consecutive intervals were included as covariates in this model.

## Results

Of the 769 parasite-positive samples, 63 were missing or did not have enough material for successful PCR amplification. With respect to the Th regions, DNA was successfully amplified and sequenced from 684 (97%) of the samples. Repeat region PCR products and fragment length data were successfully generated for 504 (74%) of the samples. Of these 504 samples, 157 were successfully sequenced via Sanger sequencing.

### Th region results

A total of 31 and 14 nonsynonymous SNPs were detected by 454 sequencing with an average coverage of ∼600x (range of 100x–1,300x) in Th2R and Th3R, respectively (Table 1). There were 17 polymorphic amino acid positions in total between the Th regions.

**Table 1:** Haplotypes and polyclonal infections detected in Th2R, Th3R and the repeat region.

	Number of haplotypes	Polyclonal infections
Th2R	87	34.9%
Th3R	23	29.8%
Repeat region	20	16.9%

The number of unique haplotypes found in Th2R and Th3R respectively was 87 and 23 (Table 1). Genbank accession numbers for the nucleotide sequence of the region containing both Th2R and Th3R are JN849502–JN849573. Of the haplotypes detected in Th2R, 51.7% occurred only once in the three year study period. Of those detected in Th3R, 37.5% occurred once in the three year study period.

**Figure 1:**
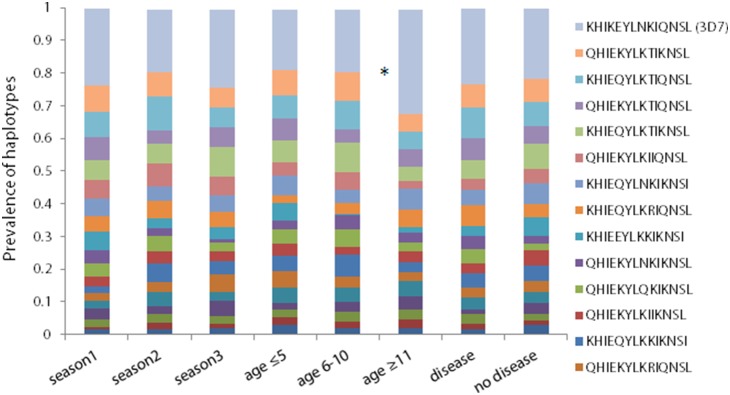
Distribution of Th2R haplotypes across seasons, age groups, and clinical and non-clinical *Plasmodium falciparum* infections. The prevalence of each unique Th2R haplotype that was present in at least 10% of all 454 reads of a given sample was calculated and stratified by the malaria transmission season in which they were collected, by three age groups (age≤5, age 6–10, and age≥11), and whether or not they were derived from samples that came from clinical vs. asymptomatic infections. Haplotype distributions did not vary significantly by age group, study year, or presence of clinical symptom for either Th2R or Th3R, with the exception of the 3D7 haplotype, which had a significantly greater prevalence in the oldest age group compared to the youngest age group (p<0.03).

**Figure 2:**
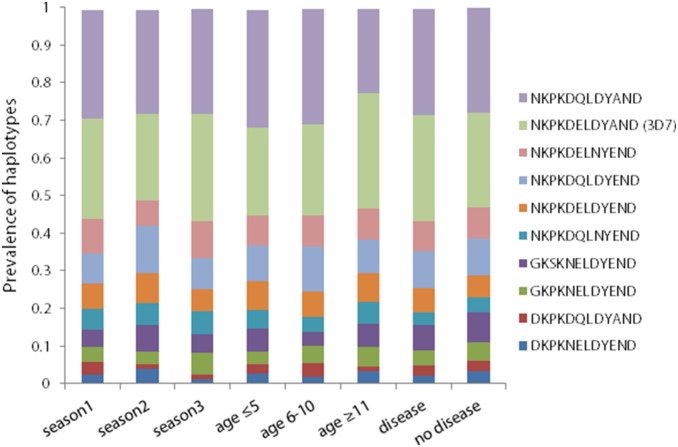
Distribution of Th3R haplotypes across seasons, age groups, and clinical and non-clinical *Plasmodium falciparum* infections. The prevalence of each unique Th3R haplotype that was present in at least 10% of all 454 reads of a given sample was calculated and stratified by the malaria transmission season in which they were collected, by three age groups (age≤5, age 6–10, and age≥11), and whether or not they were derived from samples that came from clinical vs. asymptomatic infections.

For the purposes of this study, polyclonal infections were defined as samples that contained no clear majority allele (no allele that was present in 71% or more of 454 reads). With respect to Th2R, 34.9% of samples were mixed, and with respect to Th3R, 29.8% of samples were mixed. Haplotype distributions did not vary significantly by age group, study year, or presence of clinical symptom for either Th2R or Th3R, with the exception of the Th2R 3D7 haplotype, which had a significantly greater prevalence in the oldest age group compared to the youngest age group (p<0.03, Figures 1 and 2).

### Repeat region results

**Figure 3:**
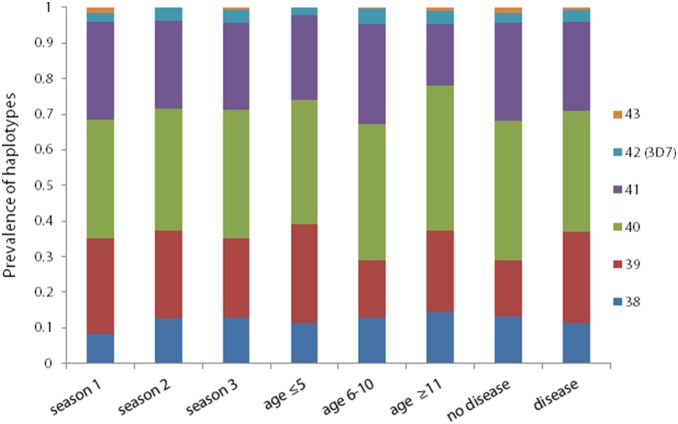
Distribution of repeat region size polymorphisms across season, age groups and clinical and non-clinical *Plasmodium falciparum* infections. The prevalence of size polymorphisms detected in the repeat region was calculated from high resolution gel analysis of the number of repeats found in this region from individual samples. Prevalences of repeat polymorphisms were stratified by the malaria transmission season in which they were collected, by three age groups (age≤5, age 6–10, and age≥11), and whether or not they were derived from samples that came from clinical vs. asymptomatic infections.

The prevalence of different repeat region sizes did not vary significantly by age group, study year, or presence of clinical symptoms (Figure 3). The most common tetrameric repeat number found in this study was 40 (34.9%), (range of 38 to 43), with the two highest numbers of repeats, 42 and 43, appearing least frequently (3.4% and 0.68%).

**Figure 4:**
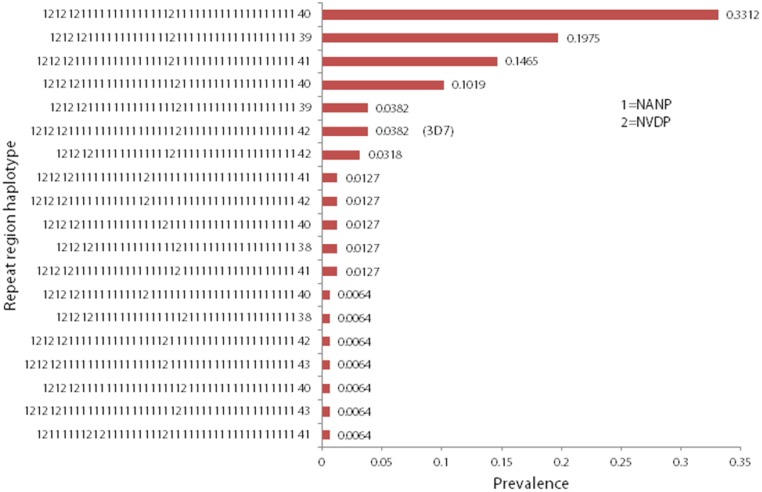
Repeat region haplotype prevalences. The prevalence of each unique repeat region haplotype that was detected by direct sequencing of ‘single clone’ (no secondary allele present in a frequency greater than 20% in 454 reads) with respect to Th2R and Th3R was calculated.

Among the 157 samples for which Sanger sequencing data were available, 19 unique haplotypes were detected, of which six were detected only once in the three year study period. Genbank accession numbers for the nucleotide sequence of these haplotypes are JQ868451–JQ868469. The majority of samples (67.5%) had one of three most prevalent haplotypes (Figure 4). When Sanger sequencing results were compared to adjusted size data generated on the Qiaxcel, there was 94% agreement between the two methods.

### Cox proportional hazards models results

**Figure 5:**
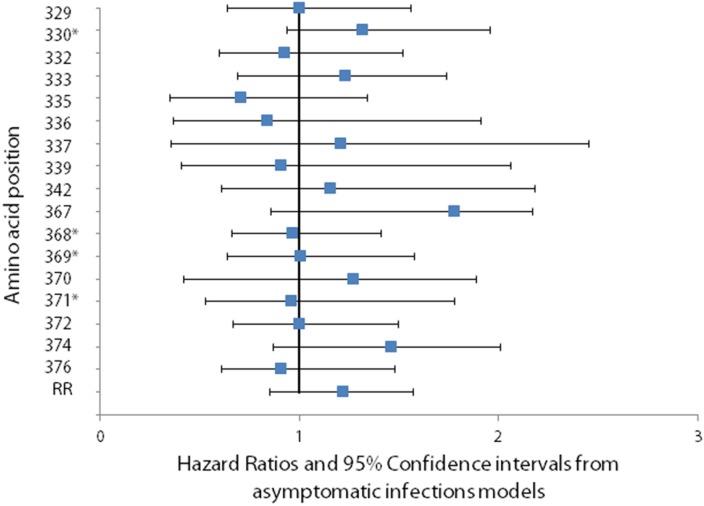
Association between change in the predominant amino at a polymorphic site and the hazard of *Plasmodium falciparum* infection. The association between changes at polymorphic sites in Th2R and Th3R which occurred between consecutive clinical episodes and the hazard of infection was calculated using a Cox proportional hazards model. To account for the possibility of treatment failure and to allow for time for allele-specific antibodies to the first of two paired consecutive infections to be present by the time of the second infection, time intervals two weeks or less between consecutive episodes were excluded from the analyses. No significant association between changes at polymorphic sites in Th2R and Th3R which occurred between consecutive clinical episodes and the hazard of infection was found. Asterisks denote polymorphic amino acid positions that lacked power to detect a hazard ratio below 1.51.

**Figure 6:**
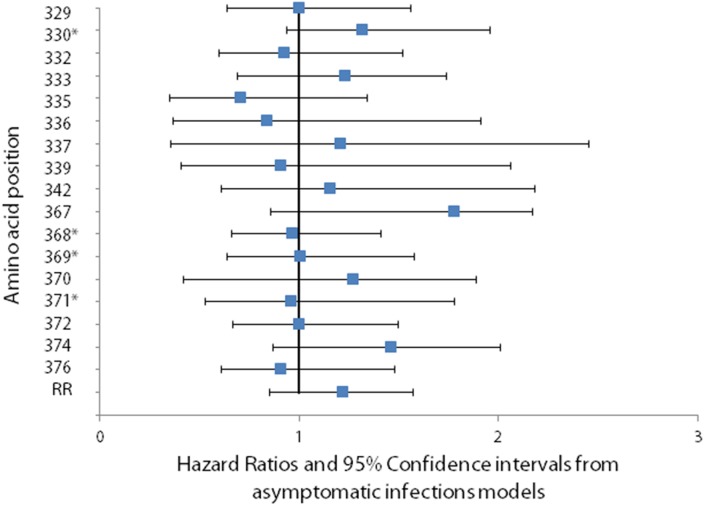
Association between change in the predominant amino at a polymorphic site and the hazard of *Plasmodium falciparum* clinical disease. The association between changes at polymorphic sites in Th2R and Th3R which occurred between consecutive clinical episodes and the hazard of clinical disease was calculated using a Cox proportional hazards model. To account for the possibility of treatment failure and to allow for time for allele-specific antibodies to the first of two paired consecutive infections to be present by the time of the second infection, time intervals two weeks or less between consecutive episodes were excluded from the analyses. No significant association between changes at polymorphic sites in Th2R and Th3R which occurred between a clinical episode and a consecutive asymptomatic infection and the hazard of clinical disease was found. Asterisks denote polymorphic amino acid positions that lacked power to detect a hazard ratio below 1.51.

There was no significant change in hazard of infection or clinical disease when a change occurred at any of the 17 polymorphic amino acid positions identified in Th2R and Th3R, or when a change occurred in the size of the repeat region (Figures 5 and 6). The number of changes between consecutive episodes was also not significantly associated with hazard of infection or disease.

### Logistic regression models results

**Figure 7:**
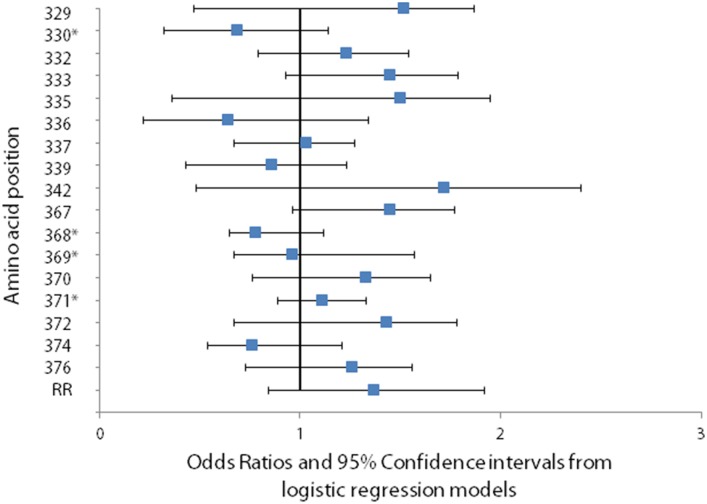
Association between change in the predominant amino at a polymorphic site and the odds of *Plasmodium falciparum* clinical disease. The odds of a change in the predominant amino acid at polymorphic residues in intervals including an asymptomatic episode followed by a symptomatic one, compared to intervals including consecutive asymptomatic episodes was calculated in a logistic regression model.

There was no significant difference in the odds of a change occurring between an asymptomatic episode followed by a symptomatic one compared to consecutive asymptomatic episodes, taking into account age and length of interval between episodes, for either the Th regions or the repeat region (Figure 7). Four of the 17 polymorphic amino acid positions in the Th region had too few observations to model.

## Discussion

Molecular epidemiological studies are an important tool for evaluating the potential of polymorphic malaria vaccine antigens to provide strain-transcending protective efficacy [1]. In this study we found no significant associations between within-host dynamics of CSP diversity and the risk of clinical disease or infections. These results imply that, in contrast to malaria blood stage antigens, immunity to this sporozoite protein may not be allele-specific, and suggest that the efficacy of vaccines based on CSP would not be improved by adding CSP antigens derived from different strains.

By using 454, this study was able to include samples containing more than one parasite clone, circumventing a problem that previous studies evaluating the allele-specificity of immunity to CSP have faced. Complete sequence reads were available for each parasite type detected by 454 so haplotypes could be constructed. In 684 samples, 87 haplotypes were found in Th2R, whereas in earlier studies using Sanger sequencing on samples from other West African settings with similar malaria epidemiology, 24 haplotypes were found in 44 Gambian samples [10], and 42 haplotypes were found for Th2R and Th3R combined in 99 samples from Sierra Leone [12]. Our data likely represent a plateauing of the total amount of diversity present in the Th regions of CSP.

Repeat region size determination was very accurate, with 94% agreement between lengths obtained by direct sequencing and those determined by high-resolution gel analysis. Using this method made it possible to examine the association between repeat region size and increased risk of infection and disease. Although the number of samples successfully sequenced was limited, results from one-third of parasite positive samples showed that 67% of successfully sequenced samples had one of three haplotypes with different lengths. This finding suggests that length polymorphism may serve as a reasonable surrogate for haplotype in the Cox and logistic regression models.

This study had some limitations which may have affected the results. Due to a large degree of polymorphism in the ThRs and a limited sample size, smaller changes in the hazard ratios between consecutive episodes in which there was a change versus no change for both clinical disease and infection could have been missed. For seven of the 23 polymorphic sites that were modeled there may not have been enough observations to fully examine associations with infection and disease. Additionally, effects of sequence variation on hazard of clinical disease or infection were not examined with respect to the repeat region due to the limited number of sequences generated. Finally, determination of polyclonal infections for the repeat region was done by capillary gel analysis, which may have been less sensitive than sequence analysis.

The large number of SNPs and haplotypes in our study is consistent with this region of the *cs* gene being under diversifying selection [25]. Although no definitive evidence has been reported of selection of non-vaccine variants of CSP following immunization with CSP-based vaccines, this idea supports the notion that genetic variation in CSP may be driven by the human immune system, implying that naturally acquired and vaccine-induced immunity may be at least to some degree allele-specific. However, the present study as well as follow-up studies to RTS,S/AS01 vaccine trials [20], [26] all suggest that immunity to CSP may not be allele-specific, in sharp contrast to polymorphic blood stage vaccine antigens such as merozoite surface protein 1 and apical membrane antigen 1 [2], [4].

If the diversity in immunogenic regions in CSP is not the target of allele-specific immune responses, the question of what is driving this diversity remains to be answered. It has been proposed that diversity in the repeat region might have evolved as an immune evasion mechanism by the parasite in which the host mounts a non-protective immune response against this region while the parasite escapes into the liver [27]. With respect to the variation in Th regions, the theory that these SNPs may result from adaptation to the salivary glands of mosquitoes of different species has been proposed [28]. The immunological phenomenon of altered peptide ligand antagonism has also been considered as a possible explanation for diversity in the Th regions [29]. In this process, competing non-protective epitopes in the Th regions may distract Th cells from recognizing the protective epitopes. Related studies have noted that recognition of T-cell epitopes within CSP can be restricted by an individual’s human leukocyte antigen (HLA) type, and individuals with certain HLA types are able to recognize a broader variety of the polymorphic epitopes than others [30], [31]. This means individuals with certain HLA types may be better equipped to fight off infection or disease caused by *falciparum* parasites. Combined molecular-immuno-epidemiological studies may help to assess these possibilities.
