# Correction: Variation in the Circumsporozoite Protein of *Plasmodium falciparum*: Vaccine Development Implications

**DOI:** 10.1371/journal.pone.0148240

**Published:** 2016-01-28

**Authors:** Kavita Gandhi, Mahamadou A. Thera, Drissa Coulibaly, Karim Traoré, Ando B. Guindo, Amed Ouattara, Shannon Takala-Harrison, Andrea A. Berry, Ogobara K. Doumbo, Christopher V. Plowe

Fig 5 is erroneously a duplicate of Fig 6. Please view the correct Fig 5 below. Additionally, the authors have provided revised captions for Fig 5 and Fig 6. Please view the corrected figure captions here.

**Fig 5:**
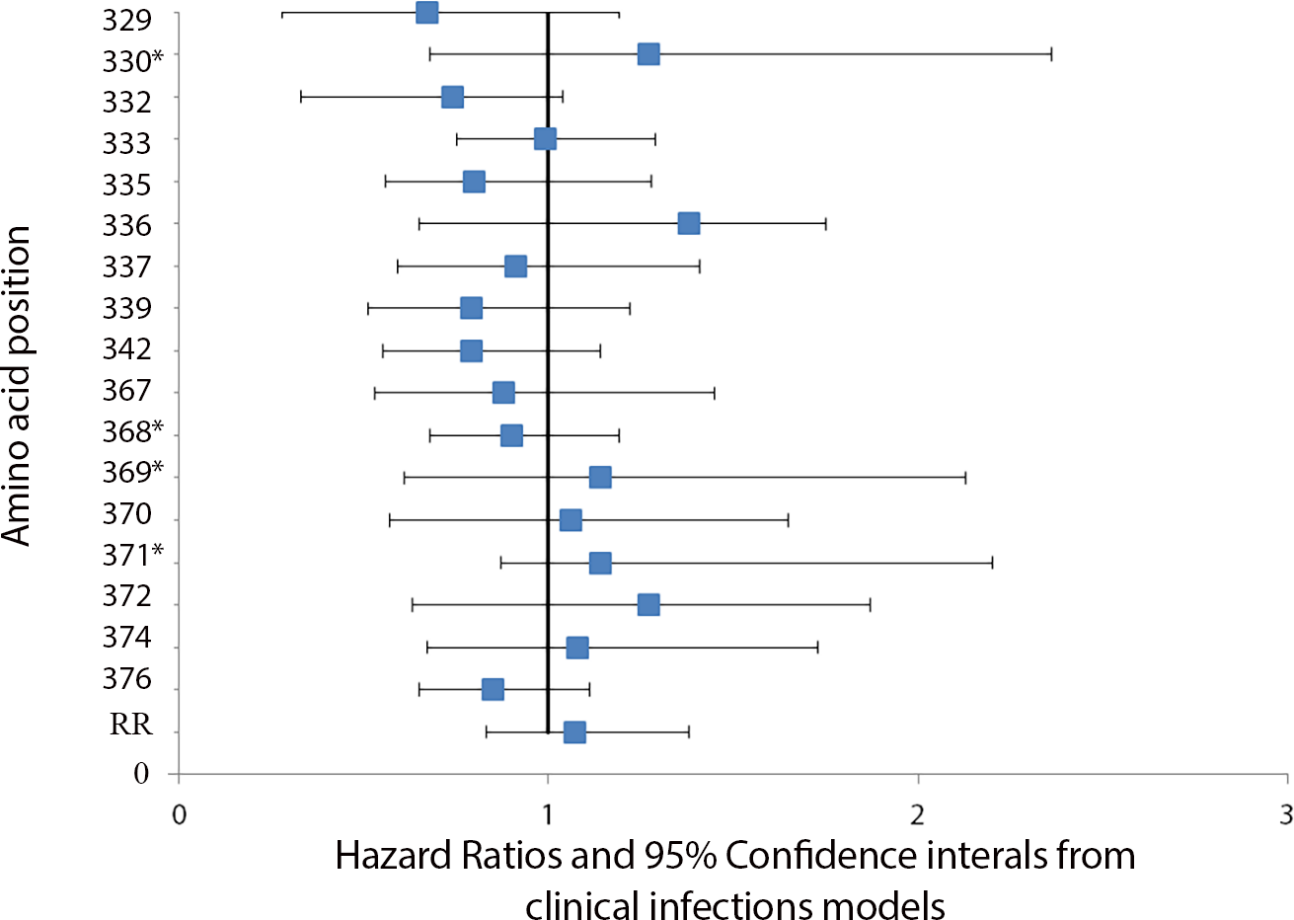
Association between change in the predominant amino at a polymorphic site and the hazard of *Plasmodium falciparum* clinical disease. The association between changes at polymorphic sites in Th2R and Th3R which occurred between consecutive clinical episodes and the hazard of clinical disease was calculated using a Cox proportional hazards model. To account for the possibility of treatment failure and to allow for time for allele-specific antibodies to the first of two paired consecutive infections to be present by the time of the second infection, time intervals two weeks or less between consecutive episodes were excluded from the analyses. No significant association between changes at polymorphic sites in Th2R and Th3R which occurred between a clinical episode and a consecutive asymptomatic infection and the hazard of clinical disease was found. Asterisks denote polymorphic amino acid positions that lacked power to detect a hazard ratio below 1.51.

**Fig 6:**
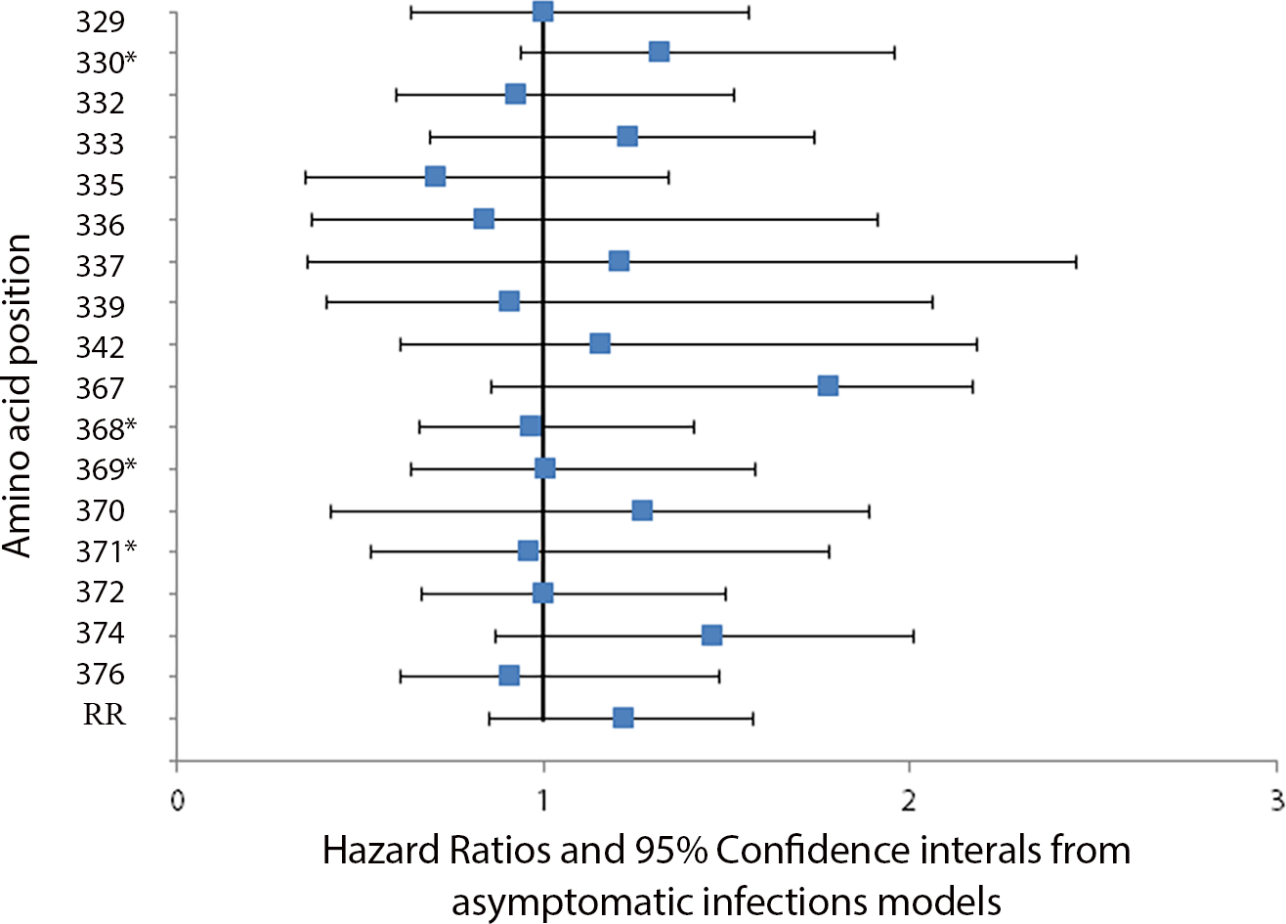
Association between change in the predominant amino at a polymorphic site and the hazard of *Plasmodium falciparum* infection. The association between changes at polymorphic sites in Th2R and Th3R which occurred between consecutive clinical episodes and the hazard of infection was calculated using a Cox proportional hazards model. To account for the possibility of treatment failure and to allow for time for allele-specific antibodies to the first of two paired consecutive infections to be present by the time of the second infection, time intervals two weeks or less between consecutive episodes were excluded from the analyses. No significant association between changes at polymorphic sites in Th2R and Th3R which occurred between consecutive clinical episodes and the hazard of infection was found. Asterisks denote polymorphic amino acid positions that lacked power to detect a hazard ratio below 1.51.
